# Release of Antibiotic Resistant Bacteria by a Waste Treatment Plant from Romania

**DOI:** 10.1264/jsme2.ME17016

**Published:** 2017-09-27

**Authors:** Iulia Lupan, Rahela Carpa, Andreea Oltean, Beatrice Simona Kelemen, Octavian Popescu

**Affiliations:** 1 Institute for Interdisciplinary Research in Bio-Nano-Sciences, Molecular Biology Center, Babes-Bolyai-University Treboniu Laurian Street 42, Cluj-Napoca RO-400271 Romania; 2 Babeş Bolyai University, Faculty of Biology and Geology, Department of Molecular Biology and Biotechnology M. Kogalniceanu Street 1, Cluj-Napoca, 400084 Romania

**Keywords:** antibiotic-resistant bacteria, wastewater treatment, removal rate, antibiotic-resistant genes, bacterial pollution sources

## Abstract

The occurrence and spread of bacterial antibiotic resistance are subjects of great interest, and the role of wastewater treatment plants has been attracting particular interest. These stations are a reservoir of bacteria, have a large range of organic and inorganic substances, and the amount of bacteria released into the environment is very high. The main purpose of the present study was to assess the removal degree of bacteria with resistance to antibiotics and identify the contribution of a wastewater treatment plant to the microbiota of Someşul Mic river water in Cluj county. The resistance to sulfamethoxazole and tetracycline and some of their representative resistance genes: *sul1*, *tet*(O), and *tet*(W) were assessed in this study. The results obtained showed that bacteria resistant to sulphonamides were more abundant than those resistant to tetracycline. The concentration of bacteria with antibiotic resistance changed after the treatment, namely, bacteria resistant to sulfamethoxazole. The removal of all bacteria and antibiotic-resistant bacteria was 98–99% and the degree of removal of bacteria resistant to tetracycline was higher than the bacteria resistant to sulfamethoxazole compared to total bacteria. The wastewater treatment plant not only contributed to elevating ARG concentrations, it also enhanced the possibility of horizontal gene transfer (HGT) by increasing the abundance of the *int*I1 gene. Even though the treatment process reduced the concentration of bacteria by two orders of magnitude, the wastewater treatment plant in Cluj-Napoca contributed to an increase in antibiotic-resistant bacteria concentrations up to 10 km downstream of its discharge in Someşul Mic river.

Bacterial resistance to antibiotics is a natural phenomenon that has been found in various environmental sites apparently free of antibiotics (24) or with trace amounts of antibiotics of a natural synthetic origin (27, 33). Even if antibiotic concentrations in the environment are not very high (in the range of μg L^−1^ or ng L^−1^) (5), together with the continuous synthesis of new antibiotics and their release into ecosystems, new mechanisms of resistance to antibiotics have increased and, thus, bacteria have developed resistance to multiple antibiotics (3).

Although antibiotic-resistant bacteria (ARB) have not emerged as a result of antibiotic discovery, a relationship has been reported between higher quantity use and the emergence of ARB (16, 29). The main antibiotic sources in the environment are unused antibiotics and their unprocessed removal, the large quantities used for veterinary purposes, the manure of birds and animals that end up in the soil and ground water, feces of human origin, and wastewater treatment plants (WWTP) (19). Antibiotic concentrations do not correlate with antibiotic resistance genes (ARG) in the environment; bacteria with antibiotic resistance already present in the environment are more important than the presence of its active components (14). Among the various sources of antibiotics in the environment, WWTP have attracted particular interest. Lactam, sulfonamide, and tetracycline resistance genes are the most frequent in wastewater (13, 17). Several factors in wastewater favor the spread of bacteria with antibiotic resistance: increased levels of antibiotics and biocides, high concentrations of resistant bacteria, and the abundance of organic and inorganic substrates (10, 19). WWTP are reservoirs of chemicals, bacteria, and genes that confer antibiotic resistance (31), and treated water releases numerous bacteria resistant to antibiotics into the environment (12, 20, 28). Some treatments of wastewater (longer hydraulic residence times) may improve the quality of treated water; however, a higher removal efficiency does not necessarily imply a significant decrease in antibiotic resistance percentages in the outflow (22).

Although the overall increase in ARB release and ARG in the environment is not a great direct risk to human health, it may increase horizontal gene transfer (HGT) and the acquirement of resistance by environmental bacteria. Due to the absence of risk assessment models for adequate evaluations of antibiotic effects on the ARB and ARG emergence, it is important to monitor the dynamics and removal grades of ARB and ARG from wastewater. Most of the studies related to this topic have highlighted the presence or absence of bacteria resistant to antibiotics, whereas fewer have reported the quantitative aspects of this matter. In the present study, ARB and ARG were assessed using traditional cultivation methods and culture-independent techniques (qPCR). Two genes for resistance to tetracycline, *tet*(O) and *tet*(W) and the sulfonamide-resistant gene (*sul1*) were used. These genes were selected to monitor the removal of bacteria with antibiotic resistance in the environment for several reasons: tetracycline and sulfonamides are widely used antibiotics that are abundant in sewage, and their quantification methods have been described previously (20).

In the present study, we also included monitoring of the integrase 1 gene (*int*I1), which is of clinical significance. Even if the gene for *int*I1 does not confer direct resistance to different classes of pollutants, it favors the acquisition and spread of multiple genes that confer resistance to various compounds. The *int*I1 gene has recently been proposed to be a marker for monitoring pollutants for various reasons: it is directly linked to the genes that confer resistance to antibiotics, chlorinated compounds, and heavy metals; it occurs in a large variety of bacteria, and its abundance varies with environmental conditions (9). The *int*I1 gene was selected because it is an indicator of exogenic pollution factors and its abundance correlates with the genes that confer resistance to antibiotics, such as the *tet* and *sul1* genes (35). The aim of the present study was to assess the abundance and extent of removal of ARB and the clinically significant *int*I1 gene at the new modern treatment plant in Cluj-Napoca, Romania.

## Materials and Methods

### Sample collection and processing

Analyses were performed on water samples from 4 sites: S1— river water 10 km upstream of WWTP; S2—entrance to WWTP; S3—discharge of WWTP; S4—river water 10 km downstream of WWTP. Samples were collected during one day in Spring and in triplicate for each site. No significant differences were observed between the abundance of ARB and ARG during seasonal periods and the highest values were observed in the Spring (37). Water was filtered through a 0.22-μm sterile filter and stored at –80°C until DNA purification.

### Physicochemical analyses

The following physicochemical parameters: pH, Eh, conductivity, O_2_ concentration, and temperature, were measured *in situ* using a portable multiparameter.

### Culture of heterotrophic bacteria

The concentrations of total bacteria and ARB were evaluated by heterotrophic plate counts. Samples were diluted and plated on R2A agar media with and without antibiotics. Resistance to two antibiotics was tested: sulfamethoxazole (50 mg L^−1^) and tetracycline (16 mg L^−1^). Plates were incubated at 25°C for 7 d.

### DNA purification

Each membrane, through which 250 mL of water was filtered (except the S2 sample, through which it was only possible to filter 100 mL), was minced by cutting and total DNA was purified using the ZR Fungal/Bacterial DNA miniprep^TM^ kit (ZymoResearch) according to the manufacturer’s instructions.

### qPCR

The primers used and sizes of the amplified fragments are shown in Table 1. PCR reactions were conducted in 20 μL containing 10 μL of SensiFAST^TM^ SYBR^®^ No-ROX mix (Bioline), 0.4 μM each of the forward and reverse primers, and 3 μL of the template. All qPCR programs consisted of initial denaturation at 95°C for 3 min followed by 40 cycles: denaturing at 95°C for 10 s, annealing for 15 s, and extension at 72°C for 15 s. All qPCR assays were performed using a Rotor-Gene 6000 machine (Corbet). Standard curves were generated using recombinant plasmids produced by the cloning genes of interest into a cloning vector. Target gene fragments were amplified by PCR using gDNA extracted from a water treatment plant. PCR products were cloned into the pJET1.2 vector (Thermo Scientific) using PCR Cloning Kit CloneJET (Thermo Scientific). Three clones from each cloning experiment were selected for sequencing. Cloned sequences were compared with the NCBI database using BLASTN. Plasmid DNA concentrations were measured using the NanoDrop spectrophotometer ND-1000. In the standard curve for all bacteria, a fragment of the 16S rRNA gene from *Escherichia coli* was amplified by PCR with the universal primers 27F (5′-TCM TGA AGA TGG GTT CTC AG-3′) (15) and 1492R (5′-ACG ACT GTT CTT GGT TAC T-3′) (34) and then cloned. The correct recombinant plasmids were used for the generation of standard curves.

Removal rate calculation

$$\text{Removal rate of ARB}=\left(1-\frac{CFU/mL\;\text{in outflow}}{CFU/mL\;\text{in inflow}}\right)\times 100\%$$

where CFU is the Colony Forming Units of ARB. Calculations of the removal rate of ARG were performed with the same equation replacing CFU with ARG.

### Statistical analysis

The Student’s *t*-test was used to test the mean values of different groups; differences were significant at a *p*-value ≤0.05. Pearson’s Correlation Coefficient was calculated in order to establish the relationship between the *int*I1 gene and ARG. SPSS Statistics was used for statistical analyses.

## Results

**Fig. 1:**
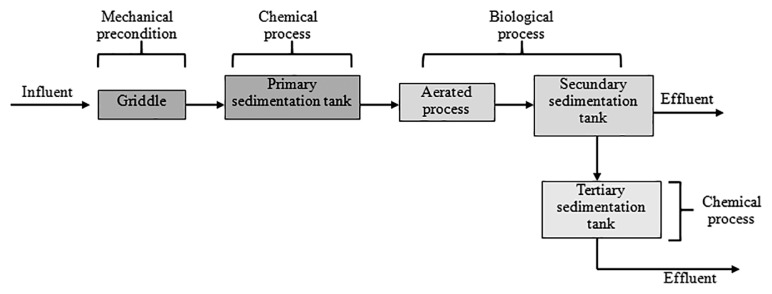
Treatment process in the Cluj-Napoca Wastewater Treatment Plant

WWTP in Cluj-Napoca is a newly modernized plant. The plant collects wastewater from a number of sources including households, hospitals, and industries. The treatment of sewage includes all 3 obligatory steps (Fig. 1) for water treatment: preliminary (mechanical removal of coarse residues); primary (chemical treatment step for the precipitation of suspensions) and secondary biological treatment stages. The treated water is discharged into Someşul Mic river. If the microbiological load is high, the water from the secondary decanter is directed to a tertiary tank for advanced treatment.

### Physicochemical analyses

Data from physical and chemical measurements of water samples are shown in Table 2. The values of dissolved oxygen in water indicated an ascending gradient from S1 (7.12) to S3 (8.96). In the S4 sample, this value was lower due to lower dissolved oxygen in river water. These values confirmed reductions in pollution during the sewage treatment.

Water conductivity was higher in S1 and S2 station samples than in S3 and S4 samples. The highest values in S2 (721 μS cm^−1^) and S1 (698 μS cm^−1^) indicate pollution that attenuates during the water treatment. This decrease was based on the removal of impurities from treated water. Water redox potential values were positive in upstream (S1) and inflow samples (S2), but negative in outflow (S3) and downstream samples (S4), with the lowest value being –76 mV in S3 and the highest value being +26 mV in S2.

pH values increased with the flow, but still remained around the neutral value; the highest pH value was measured in the S3 sample (7.21), while the lowest was in the S1 sample (6.22).

### Occurrence of ARB

**Fig. 2:**
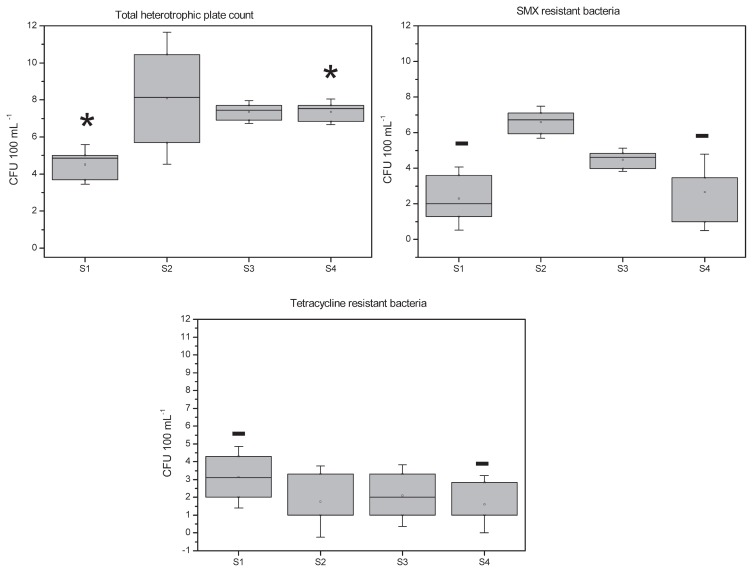
Log concentrations of total and antibiotic-resistant heterotrophic bacteria. Rectangular boxes indicate the interquartile range of data. The median value is indicated by the horizontal line inside the box. Small circles ‘q’ represent the mean values. Asterisks indicate samples that are significantly different (*p*<0.05) and the line over bars indicates samples with no significant differences (*p*>0.05).

Fig. 2 shows total cultivable bacteria as well as bacteria resistant to sulfonamide and tetracycline. Bacteria cell counts in raw water (10^8^) were 3 orders of magnitude higher than those in upstream river water (~10^5^). After the water treatment, the total number of bacteria decreased by one order of magnitude, but was still higher than that in the upstream river sample; significant differences were observed between upstream and downstream samples (*p*<0.05). The total number of bacteria in downstream river water was almost the same as that in the outflow, even 10 km from the discharge point, and no significant differences were observed between S3 and S4 samples (*p*>0.05). The treatment plant contributed to increases in total bacteria in Someşul Mic river by 2 orders of magnitude.

The number of cultivable bacteria with resistance to sulfonamide was higher in the inflow (10^7^), and this number was reduced in the outflow after the treatment by 2 orders of magnitude (10^5^). River water had the lowest concentration of bacteria resistant to sulfonamide (10^3^). Upstream and downstream samples showed similar values (*p*>0.05).

WWTP is not the only cause of bacterial resistance to antibiotics, because, unexpectedly, we found higher concentrations of bacteria resistant to tetracycline (10^3^) in the upstream sample (S1). The concentrations of tetracycline-resistant bacteria in inflow, outflow, and downstream samples were reduced by one order of magnitude (reaching 10^2^). No significant differences were observed in the concentration of tetracycline-resistant bacteria between upstream and downstream samples (*p*>0.05) (Fig. 2).

### Occurrence of ARG and the *Int*I*1* gene

**Fig. 3:**
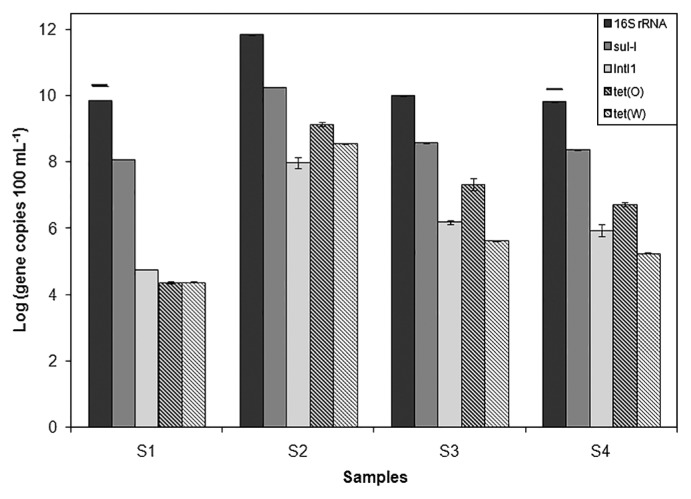
Abundance of 16S rRNA, integrase-1, and antibiotic resistance genes in water samples. Lines over bars indicate samples with no significant differences. Bars are the standard deviation of triplicate samples.

The concentration of 16S rRNA gene copies was higher in inflow water (10^12^), while similar concentrations were observed in outflow and river samples (~10^10^) (Fig. 3). Although WWTP did not increase the number of bacteria in the river, the community structure may still be affected (*p*>0.05). The upstream river sample had the lowest concentrations of ARG and the *Int*I1 gene. The concentrations of the *tet*(O) and *tet*(W) genes were the same in the upstream sample, whereas *tet*(O) was more frequent than *tet*(W) in the S2, S3, and S4 samples. The *sul1* gene concentration was higher than those of the *tet* genes in all samples, but was almost 2-fold higher in the upstream sample. The concentrations of ARG decreased by 1–2 logs after the water treatment, and were slightly lower downstream due to dilution.

**Fig. 4:**
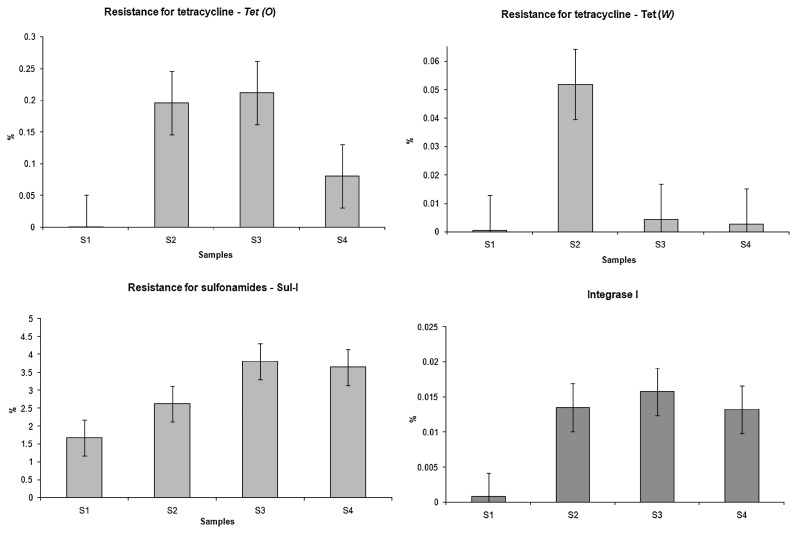
Relative concentrations of tetracycline-resistant genes (*tet*(O) and *tet*(W)), the sulfonamide-resistant gene (*sul*1), and clinically significant integrase I gene to 16S rRNA gene abundance. The bars represent standard errors.

The relative concentrations of ARG and the *Int*I1 gene to the 16S RNA gene showed a reduction in the *tet*(W) gene concentration and slight increase in the *tet*(O), *sul*-I, and *Int*I1 genes after the treatment. In all cases, the upstream sample had a lower concentration of ARG than the downstream sample (Fig. 4). Significant differences (*p*<0.05) were observed in the concentrations of the *int*I1 gene and all ARGs tested (*tet*(O), *tet*(W), and *sul1*) between upstream and downstream samples.

### Removal rate

**Fig. 5:**
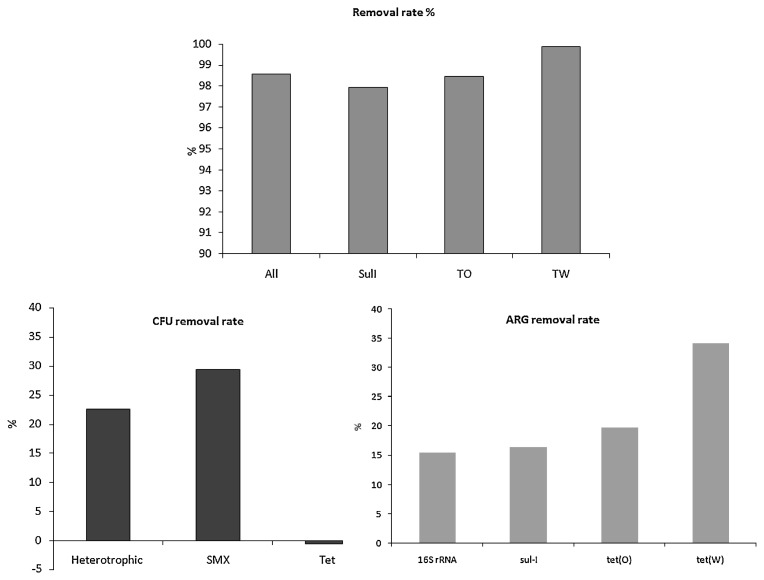
Total and log removal rates of ARB (Heterotrophic—total heterotrophic bacteria, SMX—bacteria with resistance to sulfamethoxazole, Tet—bacteria resistant to tetracycline) and ARG copy numbers.

Removal rates (the S3 to S2 ratio) for gene copies varied between 98–99% in all samples (Fig. 5A). The *tet*(W) gene was removed at higher rates than the 16S rRNA gene and *tet*(O) and *sul1* genes (Fig. 5B and C).

## Discussion

The present study focused on ARB and ARG removal rates from sewage and their release in Someşul Mic river. Although raw water had higher concentrations of ARB, their abundance was reduced compared to other studies (6, 20, 22, 36). These results may be attributed to the concentration of tetracycline tested being too high or the inability of bacteria to grow on R2A agar; bacteria in river water have a natural origin, while those in wastewater are more likely to be of a fecal origin (8). This bacterial resistance is of a natural origin; there are no other main pollution sources upstream in Someşul Mic river. Bacterial abundance in the S1 sample (10^3^) was similar to that reported previously (30). On the other hand, just one tetracycline concentration was tested and the river water and wastewater bacterial communities have different compositions (18).

Among the genes for resistance to tetracycline, *tet*(O) and *tet*(W) were the most commonly found in feces and water samples (36). Therefore, WWTP clearly contributed to the enrichment of river water with ARG and ARB (18).

Tetracycline class antibiotic concentrations in wastewater from Romania vary between 110 and 146 μg L^−1^ (23), within the limits identified in other studies, but were below the limit of detection in effluents. Even these low concentrations, in combination with heavy metals, contributed to the retention of ARB in river water (10). The relationship between the concentrations of bacteria and antibiotics has been widely studied; previous studies reported a positive correlation between the concentration of sulfonamide and bacteria with resistance to this antibiotic, but not for tetracycline (6, 17), whereas others did not (32).

Our results showed, for the first time, a strong correlation between the concentrations of the clinically significant *int*I1 gene and *sul1* gene (r=0.95), *tet*(O) gene (r=0.96), and *tet*(W) gene (r=0.96). Our results demonstrated not only the anthropogenic pollution of the river, but also a correlation between ARG and *int*I1.

The normalization of the concentrations of ARG genes tested to the 16S RNA gene suggested that the wastewater treatment increased the relative concentration of the *sul*-I gene, decreased the concentration of the *tet*(W) gene, and slightly increased the concentrations of the *tet*(O) and *Int*I1 genes (Fig. 4). These results show that the wastewater treatment lead to a decrease in the total number of bacteria, but did not necessarily reduce ARG.

The present results also indicated that not all ARG were removed at the same rate; these rates generally increased after the treatment (3, 38). ARG to classical antibiotics were more abundant in WWTP, while resistance to the latest generation of antibiotics was weaker in WWTP tanks, which constitutes a real risk to their spread and HGT (11).

Although the removal rate of bacteria was 98–99%, the small percentage discharged in the river increased bacterial concentrations and changed the microbial diversity of river water. Several studies have shown that WWTP contribute to changes in river microbiota (26).

River water downstream of the treatment plant is used for irrigation, and increases in ARB concentrations may result in the contamination of agricultural products, which are generally sold in local markets. In terms of quantity, the findings of several studies showed that ARB and ARG concentrations do not change in soils irrigated with treated water (7, 21); however, long-term irrigation may increase the relative concentrations of some ARG (4). Although irrigation with recycled water is an excellent solution to water scarcity, the possibility of strains of clinical significance reaching soil and the likelihood of finding these bacteria on vegetables, representing a higher risk for consumers, cannot be ruled out.

In conclusion the results of the present study showed that WWTP in Cluj-Napoca markedly reduced total ARG and ARB. However, WWTP contributed to an increase in ARG, even 10 km downstream of the discharge point. This increase may promote HGT, particularly because the concentrations of these genes correlate with the *int*I1 gene and also represent a human health risk due to irrigation.

In the absence of fundamental data regarding the fate of all ARB and ARG in most WWTPs and their effects on the environment and risk to public health, further studies on the extent of the removal of abundant ARB are needed in order to reach common conclusions.

## Figures and Tables

**Table 1:** Primers used for qPCR.

Target gene	Sequence (5′-3′) Forward Reverse	Amplicon size (bp)	Annealing temperature qPCR (°C)	Reference
*tet*(O)	ACGGARAGTTTATTGTATACC TGGCGTATCTATAATGTTGAC	171	50	(1)
*tet*(W)	GAGAGCCTGCTATATGCCAGC GGGCGTATCCACAATGTTAAC	168	60	(1)
rRNA 16S	AAACTCAAAKGAATTGACGG CTCACRRCACGAGCTGAC	180	60	(2)
*Int*I1	CGAACGAGTGGCGGAGGGTG TACCCGAGAGCTTGGCACCCA	312	59	(9)
*sul*1	CGCACCGGAAACATCGCTGCAC TGAAGTTCCGCCGCAAGGCTCG	163	55	(25)

**Table 2:** Physicochemical parameters of water samples

Sampling sites	pH	Eh (mV)	Conductivity (μS cm^−1^)	Dissolved oxygen (mg L^−1^)	Temperature (°C)
S1	6.22	+15	698	7.12	12
S2	6.38	+26	721	8.02	11
S3	7.21	−76	489	8.96	10.2
S4	7.06	−42	551	8.03	11

## References

[1] Aminov R.I., G-Jeanjean N., Mackie R.I. (2001). Molecular ecology of tetracycline resistance: development and validation of primers for detection of tetracycline resistance genes encoding ribosomal protection proteins. Appl Environ Microbiol.

[2] Bacchetti De Gregoris T., Aldred N., Clare A.S., Burgess J.G. (2011). Improvement of phylum- and class-specific primers for real-time PCR quantification of bacterial taxa. J Microbiol Methods.

[3] Czekalski N., Berthold T., Caucci S., Bürgmann H. (2012). Increased levels of multiresistant bacteria and resistance genes after wastewater treatment and their dissemination into lake Geneva, Switzerland. Front Microbiol.

[4] Dalkmann P., Broszat M., Siebe C., Willaschek E., Sakinc T., Huebner J., Amelung W., Grohmann E., Siemens J. (2012). Accumulation of pharmaceuticals, enterococcus, and resistance genes in soils irrigated with wastewater for zero to 100 years in central Mexico. PLoS ONE.

[5] Ding C., He J. (2010). Effect of antibiotics in the environment on microbial populations. Appl Microbiol Biotechnol.

[6] Gao P., Munir M., Xagoraraki I. (2012). Correlation of tetracycline and sulfonamide antibiotics with corresponding resistance genes and resistant bacteria in a conventional municipal wastewater treatment plant. Sci Total Environ.

[7] Gatica J., Cytryn E. (2013). Impact of treated wastewater irrigation on antibiotic resistance in the soil microbiome. Environ Sci Pollut Res.

[8] Gensberger E.T., Gössl E.M., Antonielli L., Sessitsch A., Kostić T. (2015). Effect of different heterotrophic plate count methods on the estimation of the composition of the culturable microbial community. PeerJ.

[9] Gillings M.R., Gaze W.H., Pruden A., Smalla K., Tiedje J.M., Zhu Y.G. (2015). Using the class 1 integron-integrase gene as a proxy for anthropogenic pollution. ISME J.

[10] Gullberg E., Albrecht L.M., Karlsson C., Sandegren L., Andersson D. (2014). Selection of a multidrug resistance plasmid by sublethal levels of antibiotics and heavy metals. MBio.

[11] Harnisz M. (2013). Total resistance of native bacteria as an indicator of changes in the water environment. Environmental Pollution.

[12] Jury K., Khan S.J., Vancov T., Stuetz R.M., Ashbolt N.J. (2011). Are sewage treatment plants promoting antibiotic resistance?. Crit Rev Environ Sci Technol.

[13] Kim S., Hongkeun P., Kartik Ch. (2008). The fate of tetracycline resistant bacteria in wastewater treatment plants as a function of operating characteristics.

[14] Kümmerer K. (2009). Antibiotics in the aquatic environment—a review—part II. Chemosphere.

[15] Lane D.J., Stackebrandt E., Goodfellow M. (1991). 16S/23S rRNA sequencing. Nucleic Acid Techniques in Bacterial Systematics.

[16] Levy S.B. (2002). Factors impacting on the problem of antibiotic resistance. J Antimicrob Chemother.

[17] Li D., Yu T., Zhang Y., Yang M., Li Z., Liu M., Qi R. (2010). Antibiotic resistance characteristics of environmental bacteria from an oxytetracycline production wastewater treatment plant and the receiving river. Appl Environ Microbiol.

[18] Marti E., Jofre J., Balcazar J.L. (2013). Prevalence of antibiotic resistance genes and bacterial community composition in a river influenced by a wastewater treatment plant. PLoS One.

[19] Martinez J.L. (2009). Environmental pollution by antibiotics and antibiotic resistance determinants. Environ Pollut.

[20] Munir M., Wong K., Xagoraraki I. (2011). Release of antibiotic resistant bacteria and genes in the effluent and biosolids of five wastewater utilities in Michigan. Water Res.

[21] Negreanu Y., Pasternak Z., Jurkevitch E., Cytryn E. (2012). Impact of treated wastewater irrigation on antibiotic resistance in agricultural soils. Environ Sci Technol.

[22] Novo A., Manaia C.M. (2010). Factors influencing antibiotic resistance burden in municipal wastewater treatment plants. Appl Microbiol Biotechnol.

[23] Opriş O., Soran M.L., Coman V., Copaciu F., Ristoiu D. (2013). Determination of some frequently used antibiotics in waste waters using solid phase extraction followed by high performance liquid chromatography with diode array and mass spectrometry detection. Cent Eur J Chem.

[24] Pallecchi L., Lucchetti C., Bartoloni A., Bartalesi F., Mantella A., Gamboa H., Carattoli A., Paradisi F., Rossolini G.M. (2007). Population structure and resistance genes in antibiotic-resistant bacteria from a remote community with minimal antibiotic exposure. Antimicrob Agents Chemother.

[25] Pei R., Kim S.C., Carlson K.H., Pruden A. (2006). Effect of river landscape on the sediment concentrations of antibiotics and corresponding antibiotic resistance genes (ARG). Water Res.

[26] Proia L., von Schille D., Sanchez-Melsio A., Sabater S., Borrego C.M., Rodríguez-Mozaz S., Balcázar J.L. (2016). Occurrence and persistence of antibiotic resistance genes in river biofilms after wastewater inputs in small rivers. Environ Pollution.

[27] Raaijmakers J.M., Mazzola M. (2012). Diversity and natural functions of antibiotics produced by beneficial and plant pathogenic bacteria. Annu Rev Phytopathol.

[28] Rizzo L., Manaia C., Merlin C., Schwartz T., Dagot C., Ploy M.C., Michael I., Fatta-Kassinos D. (2013). Urban wastewater treatment plants as hotspots for antibiotic resistant bacteria and genes spread into the environment: a review. Sci Total Environ.

[29] Seveno N.A., Kallifidas D., Smalla K., van Elsas J.D., Collard J.M., Karagouni A.D., Wellington E.M.H. (2002). Occurrence and reservoirs of antibiotic resistance genes in the environment. Rev Med Microbiol.

[30] Suzuki S., Ogo M., Koike T., Takada H., Newman B. (2015). Sulfonamide and tetracycline resistance genes in total- and culturable-bacterial assemblages in South African aquatic environments. Front Microbiol.

[31] Szczepanowski R., Linke B., Krahn I., Gartemann K.H., Gützkow T., Eichler W., Pühler A., Schlüter A. (2009). Detection of 140 clinically relevant antibiotic-resistance genes in the plasmid metagenome of wastewater treatment plant bacteria showing reduced susceptibility to selected antibiotics. Microbiology.

[32] Takasu H., Suzuki S., Reungsang A., Pham H.V. (2011). Fluoroquinolone (FQ) contamination does not correlate with occurrence of FQ-resistant bacteria in aquatic environments of Vietnam and Thailand. Microbes Environ.

[33] Tawiah A.A., Gbedema S.Y., Adu F., Boamah V.E., Annan K. (2012). Antibiotic producing microorganisms from River Wiwi, Lake Bosomtwe and the Gulf of Guinea at Doakor Sea Beach, Ghana. BMC Microbiol.

[34] Turner S., Pryer K.M., Miao V.P., Palmer J.D. (1999). Investigating deep phylogenetic relationships among cyanobacteria and plastids by small subunit rRNA sequence analysis. J Eukaryot Microbiol.

[35] Wang F.H., Qiao M., Lv Z.E., Guo G.X., Jia Y., Su Y.H., Zhu Y.G. (2014). Impact of reclaimed water irrigation on antibiotic resistance in public parks, Beijing, China. Environ Pollut.

[36] Yang H., Byelashov O.A., Geornaras I., Goodridge L.D., Nightingale K.K., Belk K.E., Smith G.C., Sofos J.N. (2010). Presence of antibiotic-resistant commensal bacteria in samples from agricultural, city, and national park environments evaluated by standard culture and real-time PCR methods. Can J Microbiol.

[37] Yuan Q.B., Guo M.T., Yang J. (2014). Monitoring and assessing the impact of wastewater treatment on release of both antibiotic-resistant bacteria and their typical genes in a Chinese municipal wastewater treatment plant. Environ Sci Processes Impacts.

[38] Zhang Y., Marrs C.F., Simon C., Xi C. (2009). Wastewater treatment contributes to selective increase of antibiotic resistance among *Acinetobacter* spp. Sci Total Environ.

